# Providing open imaging data at scale: An EMBL-EBI perspective

**DOI:** 10.1007/s00418-023-02216-2

**Published:** 2023-08-03

**Authors:** Matthew Hartley, Andrii Iudin, Ardan Padwardhan, Ugis Sarkans, Aybüke Küpcü Yoldaş, Gerard J. Kleywegt

**Affiliations:** European Molecular Biology Laboratory, European Bioinformatics Institute (EMBL-EBI), Hinxton, UK

**Keywords:** Bioimaging, Data management, Data integration, Metadata

## Abstract

Biological imaging is one of the primary tools by which we understand living systems across scales from atoms to organisms. Rapid advances in imaging technology have increased both the spatial and temporal resolutions at which we examine those systems, as well as enabling visualisation of larger tissue volumes. These advances have huge potential but also generate ever increasing amounts of imaging data that must be stored and analysed. Public image repositories provide a critical scientific service through open data provision, supporting reproducibility of scientific results, access to reference imaging datasets and reuse of data for new scientific discovery and acceleration of image analysis methods development. The scale and scope of imaging data provides both challenges and opportunities for open sharing of image data. In this article, we provide a perspective influenced by decades of provision of open data resources for biological information, suggesting areas to focus on and a path towards global interoperability.

## Introduction

Biological imaging has a long history as a key approach for understanding biological systems. From early microscopy with hand-ground lenses through to modern, highly automated microscopes, imaging technology has developed hand in hand with our understanding of life itself. Since the dawn of computational microscopy, improved sensor technology, automation and scaling have enabled ever increasing volumes of image data from many different modalities to be collected.

Not only are data volumes increasing rapidly, but deriving scientific results from imaging data increasingly requires complex analysis pipelines that act on raw images to eventually produce information (Bagheri et al. 2022). These pipelines themselves are also undergoing rapid development, with artificial intelligence (AI) approaches radically changing biological image analysis (von Chamier et al. 2019).

Besides this growth in data volumes, there has been increasing community pressure to make imaging data publicly available, both to support reproducibility and to enable new research using existing data.

### The benefits of open data sharing

Open sharing of image data has several benefits, particularly when data are shared according to the findable, accessible, interoperable, and reusable (FAIR) principles (Wilkinson et al. 2016). These benefits include the following:

- It supports reproducibility of the analyses that underlie scientific manuscripts by providing access to the raw and derived images that give rise to quantitative results, particularly when image data is linked to the analysis methods used to derive those results.
- Over time, it allows the construction of collections of curated data that provide community standard references for particular domains of imaging or biological study. Examples include cell or tissue atlases.
- It reduces the need for replication of imaging experiments, saving time, reducing cost and reducing the need for using animals in research.
- It accelerates development and improvement of image analysis methods through the provision of data for training, validation and testing of new approaches.

Beyond the benefits to the scientific community, open data sharing provides benefits to individual researchers. These include increased visibility of work and routes towards citation and re-use that are different from primary manuscript publication (Piwowar and Vision 2013).

### EMPIAR and the BioImage Archive: background

The European Molecular Biology Laboratory’s European Bioinformatics Institute (EMBL-EBI) provides public data resources for many domains of biological research. This includes biological imaging, with the Electron Microscopy Public Image Archive (EMPIAR, https://www.ebi.ac.uk/empiar/) and the BioImage Archive (BIA, https://www.ebi.ac.uk/bioimage-archive/) together providing free public archival of imaging data from any modality, at any scale.

EMPIAR (Iudin et al. 2023) was established in 2013, initially to hold raw images underlying electron cryo-microscopy (cryo-EM) volumes used for structural biology, with the maps and tomograms themselves deposited in the Electron Microscopy Data Bank (EMDB; Lawson et al. 2016) and any atomic models in the Protein Data Bank (PDB; wwPDB Consortium 2019). Driven by community demand, EMPIAR’s scope has since expanded to include volume electron microscopy (vEM) (Peddie et al. 2022), as well as soft and hard X-ray tomography.

The BioImage Archive (Hartley et al. 2022) was launched in 2019, following a community call for the provision of a general-purpose repository for biological imaging data (Ellenberg et al. 2018). The BIA accepts data from any imaging modality that does not have a more specialised resource available (e.g. EMPIAR for 3D electron microscopy data), and builds on the generalist BioStudies resource (Sarkans et al. 2018). It acts as a deposition database, with the intent to support added-value resources such as the Image Data Resource (Williams et al. 2017), which provides highly curated reference imaging datasets.

### Data growth and diversity

**Fig. 1:**
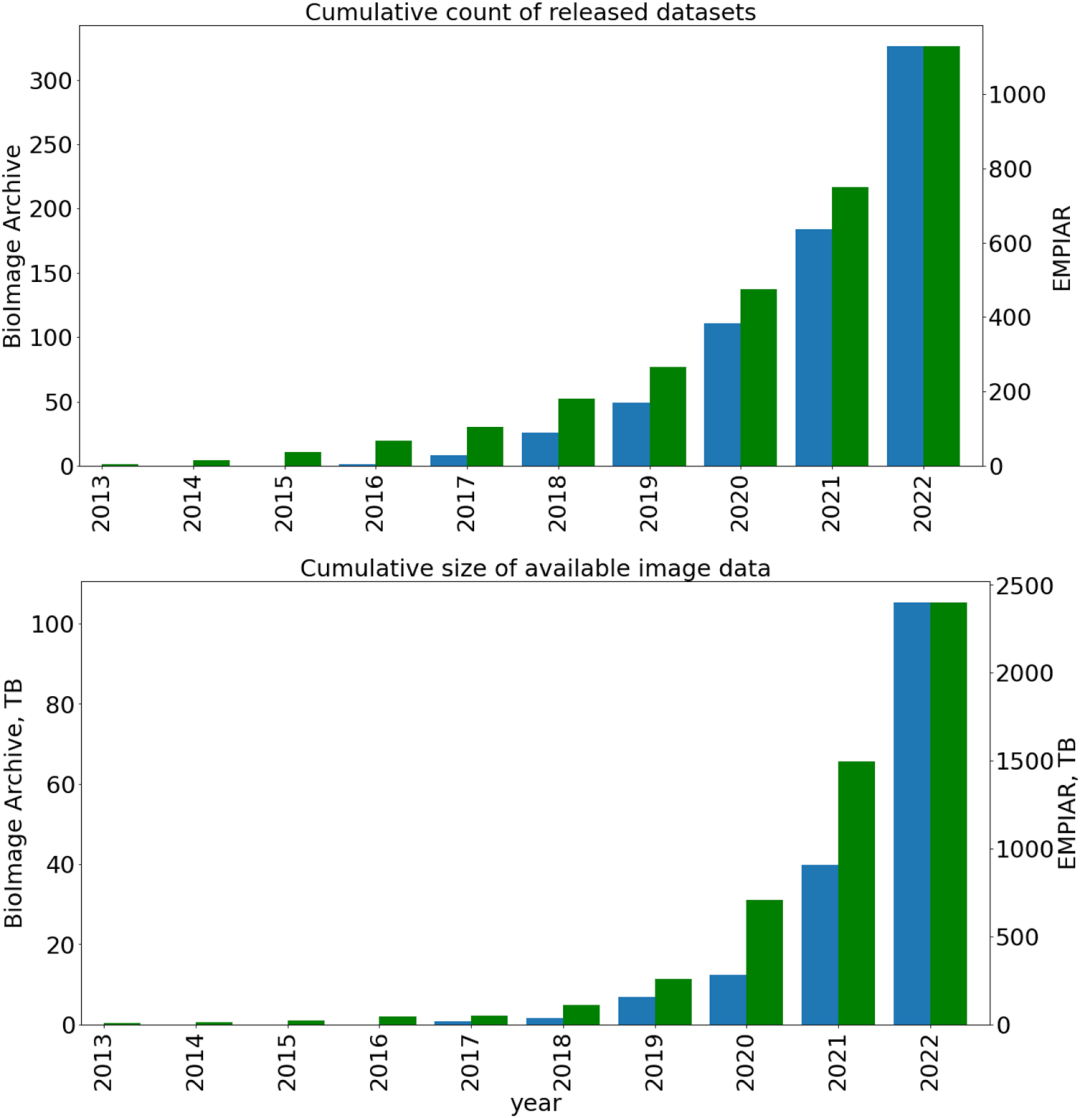
Growth in the total number of released datasets (top panel), and cumulative size of available image data (bottom panel) for EMPIAR (green) and the BioImage Archive (blue)

Both EMPIAR and the BIA have grown rapidly since their initial launches (Fig. 1). At the time of writing (May 2023), the two resources combined make nearly 3 petabytes of imaging data openly accessible to the scientific community.

Beyond sheer scale, the range of imaging modalities represented in the archives’ collections has also grown rapidly. The BioImage Archive’s generalist role is represented with imaging data from over 20 different imaging modalities currently available for reuse. While EMPIAR’s primary initial focus was cryo-EM, adding the many techniques that together comprise vEM has increased the diversity of imaging types considerably.

#### Box 1: EMBL-EBI data resources

EMBL-EBI’s data resources support a wide range of biological domains; see Table 1 below for examples. These span resources that are (a) extremely large in data volume [including those that hold 100 s of petabytes of data such as the European Nucleotide Archive (ENA) and European Genome–Phenome Archive (EGA)]; (b) long lived [such as the Protein Bank in Europe (PDBe), part of the Worldwide Protein Data Bank (wwPDB), a resource founded in 1971 and now over 50 years old); and (c) unique in their domain [such as the genome-wide association studies(GWAS) Catalog].

**Table 1:** EMBL-EBI data domains and example resources

Domain	Example resources
Chemicals, molecules and drug discovery	ChEMBL, MetaboLights, Open Targets
Genes, genomes and RNA	ArrayExpress, Ensembl, European Nucleotide Archive, Rfam (Rfam), Mgnify
Proteins	Protein Data Bank in Europe (PDBe), Proteomics Identification Database (PRIDE), UniProt
Imaging	BioImage Archive, Electron Microscopy Databank, EMPIAR
Genetic variation and disease	Coronavirus disease 2019 (COVID-19) data platform, European Genome–Phenome Archive (EGA), European Variation Archive (EVA)
Literature and knowledge management	BioSamples, BioStudies, Europe PubMed Central (PMC), GWAS Catalog, Ontologies

These resources receive over 3 billion web requests per month, from over 5 million unique internet protocol (IP) addresses. They are used across the globe, with access in 2021 recorded from every United Nations (UN) member state country. Many resources are leading members of international consortia such as the International Nucleotide Sequencing Consortium, wwPDB or the ProteomeXchange Consortium. Together, they provide immense value to scientists worldwide.

## FAIRness at scale: challenges

Providing access to imaging data at this scale reveals many challenges, some of which are unique to the imaging world, and others that are more general.

### Biological imaging is heterogeneous

**Fig. 2:**
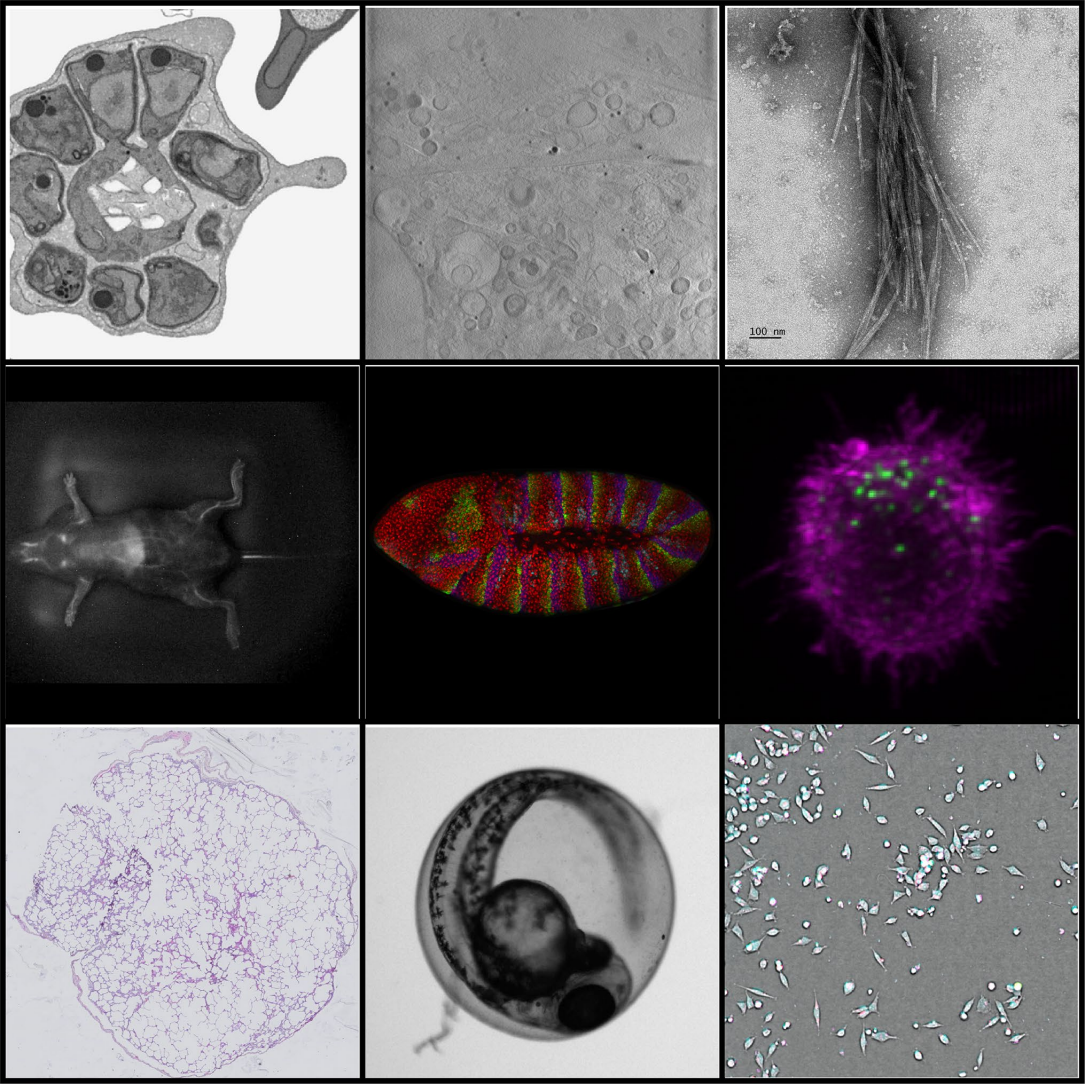
Diversity in imaging applications/modalities. From left to right, top to bottom: focussed ion beam scanning electron microscopes (FIB-SEM; EMPIAR-10310), soft X-ray tomography (EMPIAR-10416), 2D electron microscopy (S-BIAD462), shortwave infrared imaging (S-BIAD548), confocal fluorescence microscopy (S-BIAD582), lattice light-sheet microscopy (S-BIAD573), whole-slide imaging for histology (S-BIAD419), widefield microscopy (S-BIAD531) and multichannel high-content screening (S-BIAD145)

Biological imaging is a broad term, covering a very wide range of imaging techniques, including the many variants of light-based microscopy as well as electron microscopy, scanning probe microscopy and others (Fig. 2). Each of these technologies is essentially a field of its own, with its own community, standards, practices and approaches. They also advance rapidly, with a consequent need for new analysis tools and methods, as well as new nomenclature and metadata standards for understanding and organising the generated images.

These differences in capture methods, and their implementation in different instruments, have given rise to many different file formats in which image data can be represented. For those wishing to view, access and reuse open imaging data, this morass of file formats is a significant barrier.

In addition, public image data resources must organise these different types of images in ways that ensure they remain findable. Organising data requires good-quality metadata (Linkert et al. 2010). However, these heterogeneities in biological imaging techniques make establishing consistent metadata standards much more difficult.

### Imaging data is large on several axes

As we have shown, the volume of data provided by both EMPIAR and the BioImage Archive is growing very rapidly. This reflects a general growth in the volume of image data under management, which presents different challenges.

#### Total volume of data

As individual depositions grow in size, larger volumes of data must be transferred from the submitter to the archive (or between institutions for pre-publication management of data). EMPIAR’s largest individual datasets are over 70 terabytes in size, which can potentially take many weeks to transfer, particularly since network bandwidth is not scaling at the rate of data growth. Automated checks that run on data files (for example, to determine the integrity of images) often have to read the entire image, which scales linearly with image size. Scaling storage to cope with this data volume also requires significant financial and infrastructural commitment, although there are economies of scale that help large institutional data providers.

Very large individual images are also challenging to work with. Such large individual images arise from different capture methods – vEM techniques can produce very large images, as can techniques that stitch 2D electron microscopy images (Faas et al. 2012). Light-sheet microscopy, in particular long time series across multiple fluorescence channels, also generates extremely large individual images. These individual images can be hundreds of GBs to TBs in size. They can be difficult to work with in computer memory, and when shared, are difficult for users to access if they need to download the entire image to work with it.

Large imaging datasets are sometimes structured into extremely large numbers of individual files, either because such datasets involve multiple images captured by highly automated processes (e.g. high-throughput microscopy) or because of sectioning techniques where each section image is stored individually. These are difficult for traditional computer file systems to deal with; directories with millions of individual files cause significant performance issues for example. Such datasets also require carefully chosen ways in which to store metadata since supporting searches across millions of files can be challenging.

Finally, the number of individual datasets that must be managed is a challenge. Human curation of datasets is often required to ensure appropriate standards have been followed. Time required for this curation scales linearly with the number of datasets that must be reviewed. Additionally, as datasets become more complex, time required for curation also increases, such as for the correlative or multimodal data described below for which curation requires expertise in multiple different biological data domains.

### Image data is often integrated with other data types

Part of the increased complexity in biological imaging comes from multimodality. Imaging across scales often requires correlative approaches, for example, combining fluorescence microscopy to identify regions of interest followed by detailed exploration using electron microscopy (Sartori et al. 2007). This allows the combination of covering large sample volumes while providing high resolution where needed.

Imaging data is increasingly combined with other types of biological data such as transcriptomic, proteomic or metabolomic data. These “spatial omics” techniques allow precise physical and temporal localisation of key biological processes. However, this data integration makes consistent data standards even more critical since integration depends on identification and classification. For screening datasets, linking to knowledge bases such as ChEMBL (Gaulton et al. 2017; Mendez et al. 2019) and UniProt (The UniProt Consortium 2023) is often of additional importance for consistency of identification of compounds or proteins screened.

**Fig. 3:**
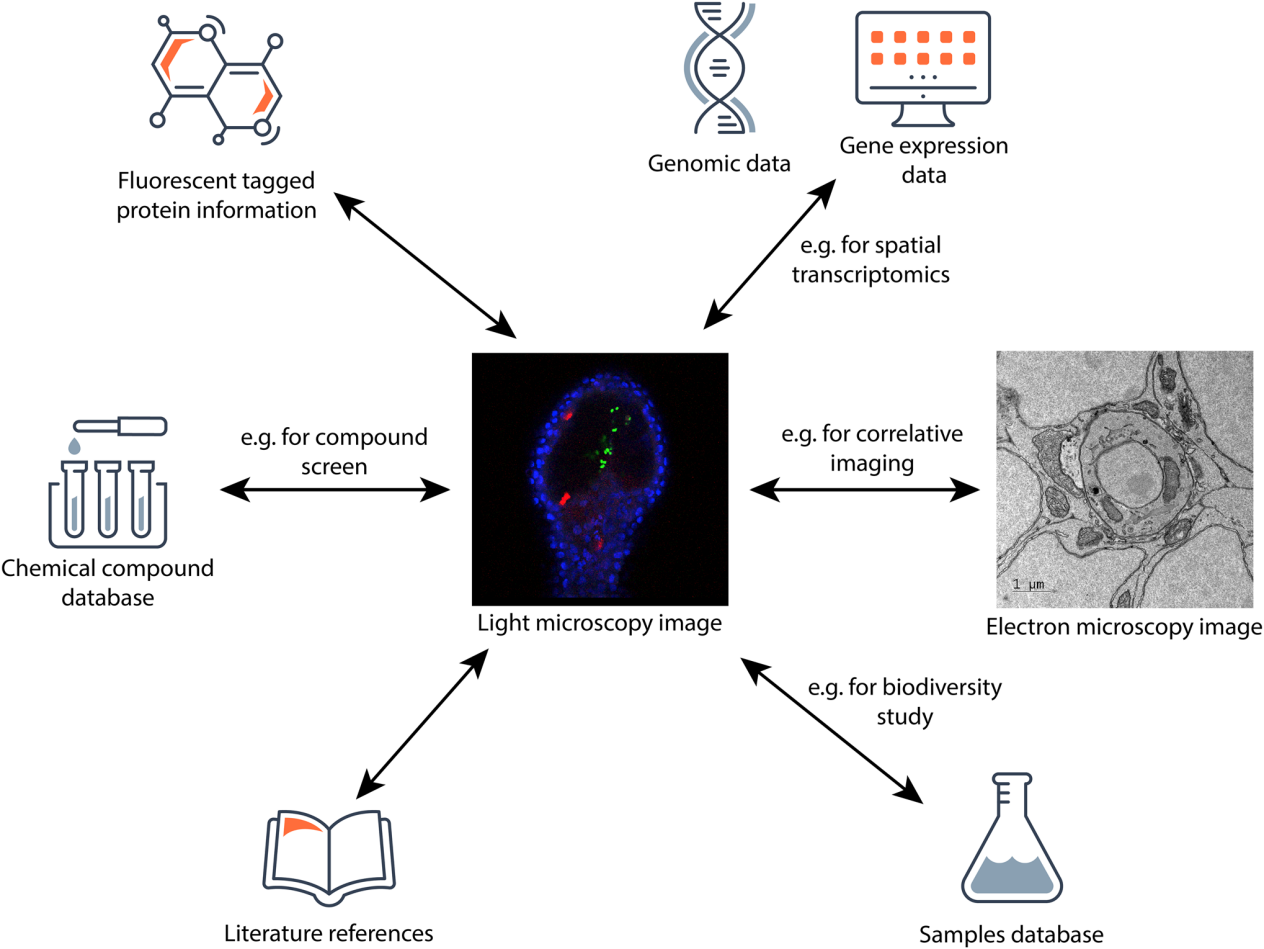
Describing an image in ways that make it useful for reproducibility and reuse often involves references and links to other types of biological information. Central image from S-BSST429, “The zebrafish as a novel model for the in vivo study of *Toxoplasma gondii* replication and interaction with macrophages” (Yoshida et al. 2020), with a corresponding image from EMPIAR-10460 at its right. Specialised data resources for these other biological domains can provide dedicated functionality, for example, BioSamples (Courtot et al. 2022) for sample management, the European Nucleotide Archive for genomic sequence data (Cummins et al. 2022) and the Flurescent Protein Database (FPBase) for fluorescent proteins (Lambert 2019)

Archiving and providing access to these datasets is also a challenge. Often archives specialise in different data types – for example, EMBL-EBI offers archives for gene expression data (Moreno et al. 2022), proteomics (Perez-Riverol et al. 2022) and metabolomics (Haug et al. 2020) individually. For multimodal data, consideration must then be given to whether and how incoming data should be split or replicated, as well as presented as a unified whole (Fig. 3).

## From challenges to opportunities

These challenges also present opportunities – more comprehensive, higher resolution imaging data tightly integrated with other biological data types has huge potential to advance our understanding of biological systems (Lewis et al. 2021). Managing the heterogeneity and scale becomes possible by focussing on consistent data standards, for both formats and metadata, together with close community involvement.

### Standardised data formats and use-appropriate storage

Mitigating the challenges of large images, particularly making them easily browsable and accessible, is possible with appropriate use of modern cloud-optimised file formats that are designed to accommodate rapid access to components of an image, and include precomputed downsampled representations to allow rapid previews. The OME-NGFF file format (Moore et al. 2021) provides all of these features, and has strong support for wide adoption (Moore et al. 2023). Hosting images in these data formats using object storage provides both a way to scale up to very large storage systems and an access protocol for data that allows structured data to be shared more easily.

Choosing data storage technologies requires making a number of trade-offs between cost, capacity, speed, redundancy/resilience, access protocols and other factors. Object storage is typically cheaper per unit of volume than filesystem storage (for example, as of May 2023, Amazon Elastic File Storage is approximately 10 times more expensive than equivalent S3 object storage). Nevertheless, data access patterns vary, with some datasets accessed much more frequently than others; hence, it is possible to tune storage solutions to access patterns. In particular, we can usefully combine faster storage for more recent and more frequently accessed datasets, with cheaper, more capacious storage for others. For rarely accessed data, images can be kept on “cold storage” such as magnetic tape. This “tiered” storage approach smooths the path towards long-term sustainability.

### A path to harmonised metadata

Key early work on image metadata developed standards and models for image files (Allan et al. 2012), generally with a focus on metadata related to the process of image acquisition and how image data is structured within files. Later extensions of this work have refined and improved the model for specific domains such as light microscopy (Hammer et al. 2021).

**Fig. 4:**
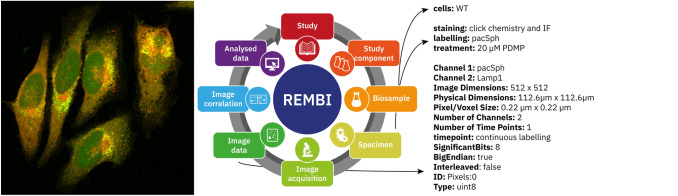
Display of an image together with metadata aligned with the REMBI model including image file structure, sample treatment and fluorescence channel content, from BioImage Archive accession S-BIAD144 (Hartwig and Höglinger 2021)

To understand the context of the results of an imaging experiment also requires information about the purpose of the experiment, samples imaged and their preparation, and how images were analysed and potentially linked to other data sources. Recommended Metadata for Biological Images (REMBI; http://bit.ly/rembi_v1) is a set of guidelines developed by representatives of different subgroups within the wider biological imaging community (Sarkans et al. 2021). It provides organising principles for imaging metadata that capture all of this context needed to enable reuse of images (Fig. 4).

Together, these detailed models and organising principles provide a route towards harmonising metadata models across different repositories for imaging data. A shared understanding of model components supports mapping of metadata and better interoperability. This is particularly important for integrating multimodal data, where using standardised and shared identifiers across data domains is vital.

### Working with the community

Like imaging itself, the biological imaging community is large and diverse. Different domains of biological imaging often have their own subcommunities, with separate networks, meetings and preferred dissemination routes. However, imaging organisations across the national, continental and international spheres such as Euro-BioImaging, BioImaging North America and Global BioImaging provide focal points for these communities to come together.

Imaging scientists have a key role in this community. They work directly with experimental biologists carrying out hypothesis-driven research, and support and develop imaging instrument technologies. Their involvement is critical to the long-term success of large-scale image data sharing. Imaging scientists should be supported through the provision of guidance, tools, training and documentation to aid the process of annotating and sharing well-curated imaging datasets. Where these scientists work to support and train others (for example, in imaging facilities), a “train-the-trainer” approach enables the widening of the reach of good practice. Furthermore, their feedback and guidance are critical to those attempting to develop user-friendly systems for submission, curation and reuse of image data.

The bioimage analysis community is a critical piece of the image data puzzle (Cimini et al. 2020). Method developers contribute to data resources through publication of their work, as well as benefiting from open access to data to test and validate their approaches. Those providing training courses often make use of public data for demonstrations. Sequence and structural data resources supported and evolved alongside the changing needs of the bioinformatics community; similarly, imaging data resources and bioimage analysts have much to offer each other.

Finally, as a wider range of shared resources for managing image data come online, there is substantial opportunity for working together effectively. Large-scale archives can work with local and national facilities to agree on standards, develop publishing pipelines and collaborate on tools and methods. As more national and international data resources emerge, there is potential to link them together to share the demands of large-scale data provision.

### From instrument to archive: pre-publication image data

Pre-publication data management, from project planning through capture and primary analysis, has somewhat different goals from public data sharing. However, the two domains have considerable overlap in both challenges and methods. In particular, the diversity of formats, gaps in metadata standardisation and sheer scale of data volumes all cause difficulties. Where data must be shared across collaborating groups or teams, the challenges of data transfer also apply.

Fortunately, well supported open-source community tools that address some of these challenges provide a starting point for institutions and facilities looking to manage their data. These tools, which include OME Remote Objects (OMERO; Allan et al. 2012), XNAT (Herrick et al. 2016) and Cytomine (Marée et al. 2016) as well as many others, provide platforms for storing, organising and visualising images, as well as managing user accounts and providing various degrees of integrated analysis software.

Beyond these ready-made solutions, standardisation of image data formats and metadata models provides opportunities for shared building blocks between pre-publication data management and public repositories. On this shared basis can be built tools for managing data movement, integrating with analysis software and data annotation/curation.

### Sensitive data: further challenges and further benefits

This manuscript’s primary focus is on sharing of image data that are not sensitive (i.e. those that do not include patient identifiable information). The challenges of sharing sensitive data compound the difficulties described above with the legal, ethical and regulatory demands on managing infrastructure for controlled access. However, there are clearly huge potential benefits to being able to share sensitive human imaging data, including the development of new clinical methods and diagnostic tools.

The European Genome–Phenome Archive (EGA; Freeberg et al. 2022) provides a service for permanent archiving and sharing of personally identifiable genetic, phenotypic and clinical data. While most current use of the EGA is for sharing of genetic data, there is potential either for co-development with imaging-focussed data resources to enable support for archival and distribution of imaging data linked to genetic data or for taking the EGA developments as a pattern for the development of image-focussed sensitive data resources. This work might perhaps initially be funded through structured data generation projects and scaled up.

## Looking to the future

### Lessons from other data domains in biology

EMBL-EBI provides data resources across many domains of biological data (Thakur et al. 2023). These include databases such as the Protein Data Bank (wwPDB Consortium 2019) that have existed for over 50 years, effectively as old as computational biology itself. Experience managing biological data resources at scale has taught several lessons with relevance to the future path for managing imaging data across the globe.

#### Importance of guidelines and standards

Use of metadata recommendations such as Minimum Information About a Microarray Experiment (MIAME) (Brazma et al. 2001) or Minimum Information about a Sequencing Experiment (MINSEQE) (Brazma et al. 2012), together with ontologies such as the Experimental Factor Ontology (Malone et al. 2010), have improved the interoperability of data, particularly allowing comparison across experiments. This is essential for large-scale reanalysis and effective sharing. Central archival resources have an important role as guardians and advocates of these standards, without which consistent enforcement is difficult. The REMBI guidelines provide a starting point to fill this role for biological imaging.

#### Simplicity

Scientific communities and projects have limited resources when it comes to supporting data exchange and publishing. Standards implementations that are initially complex (Spellman et al. 2002) have steep learning curves, but benefit from simplification over time (Rayner et al. 2006). Simpler standards provide easier, and hence wider, adoption, which in turn supports further development of those standards.

#### Separating data archiving and added-value resources

The main principles of building a successful scientific data archive are: capture experimental data and supporting metadata, i.e., scientific record; provide a simple data submission process; facilitate standards development, and adopt existing standards; and do not perform data value judgements or complex data integration. A value added resource may enrich, combine and curate datasets; reprocess data; run meta-analysis; etc. The two sets of functions are quite distinct and require different approaches to resource governance, processes and technical infrastructure.

#### Need to grow and adapt together with the global community

As data resources have developed, the need for large-scale collaborations to share the burden of management has grown. The International Nucleotide Sequence Database Collaboration (Cochrane et al. 2011) and Worldwide Protein Data Bank (Berman et al. 2003) came into being as their respective biological domains matured and the need for joint provision of resources grew. For data consumption, it is important to work with data analysts and understand what useful data access patterns need to be supported.

#### Value of data integration

The results of individual experiments do not exist in a vacuum. The systems, organisms, processes, genes, compounds, tissues and cells studied by imaging are examined by multiple routes of study. As discussed above, imaging data is often at its most informative when it is combined with other kinds of data. Ensuring that image metadata are recorded in ways that allow linking to these other data supports such data integration. An example is the use of consistent identifiers for proteins in a localisation study so that they can be linked to a protein sequence database such as UniProt (The UniProt Consortium 2023), and from there to many other resources.

#### Long-term-ism in data reuse

Beyond immediate reuse cases, providing well-organised biological data can bring unexpected benefits. The AlphaFold Protein Structure Database (Varadi et al. 2021) based on AlphaFold (Jumper et al. 2021) has revolutionised structural biology. AlphaFold builds on the years of careful curation, standards development and community encouragement for shared archiving since the PDB’s inception in 1971, and the even older UniProt antecedents (Dayhoff 1969) that precede electronic databases. At the time of this inception, the deep learning methods underlying AlphaFold’s success did not exist, and few could have predicted how successful and important they would become half a century later.

### Providing FAIR image data at scale: a path forward

Considering the possibilities enabled by cloud-ready imaging formats, the development of metadata models and the guidance from other domains of biological data, we can suggest next steps to support large-scale open sharing of image data and metadata.

#### Work towards a small set of standard image formats for distribution

As the community converges on a small set of imaging formats that can meet the needs of data generators, analysts and depositors, it becomes possible to build a rich ecosystem of tools and resources around them. In addition to reducing the need for data conversion, these formats provide a standardised protocol for online data access, mitigating the need for time-consuming downloads, and enabling rapid visualisation.

#### Agree on shared vocabularies for working with image data

Controlled vocabularies and full ontologies are key components of enabling data integration and comparison. Agreement on which of these to use across the community of those managing imaging data would be a good first step towards enabling interoperability.

#### Develop and grow shared core metadata standards

Although the needs of different data management systems differ, agreeing on a shared core set of metadata based on the REMBI principles would provide a basis for interoperability. An initial core set could be agreed across existing data providers and extended over time.

#### Align data management systems across scales

Once standardised data formats, shared vocabularies and metadata schemas are aligned, collaboration across data resources that manage imaging data becomes much easier. Data can flow between pre-publication data management systems and public repositories, and federation across large-scale archives becomes possible.

## Concluding remarks: the promise of open, FAIR imaging data

Open data is fundamental to good science. It supports reproducibility and auditability, without which science cannot prosper. It helps with method development by providing reference example data for image analysis tools and techniques. Open data is particularly critical for biological imaging because the field moves so fast, with new developments in technology allowing access to higher spatial and temporal resolutions, larger tissue volumes and more multiplexing.

A recent case study in image data reuse highlights the benefits of open provision of well-annotated image data. The Nucleome Browser (Zhu et al. 2022) is a data visualisation and exploration platform that integrates many different types of biological data to investigate nuclear structure and function. The browser uses data from the Image Data Resource (Williams et al. 2017), and relies on the annotations and curation applied to those data.

If these approaches can be enabled for the huge volumes of imaging data now captured, there are rich opportunities for examining the systems of life at higher detail and improving our understanding of biology at unprecedented scales. Taking lessons from the wider world of biological data and building a roadmap from data and metadata standards will enable realisation of this vision.

## Data Availability

All data shown in the figures is available publicly under permissive licenses. No new datasets were generated as part of this study.

## References

[CR1] Allan C, Burel J-M, Moore J (2012). OMERO: flexible, model-driven data management for experimental biology. Nat Methods.

[CR2] Bagheri N, Carpenter AE, Lundberg E (2022). The new era of quantitative cell imaging – challenges and opportunities. Mol Cell.

[CR3] Berman H, Henrick K, Nakamura H (2003). Announcing the worldwide Protein Data Bank. Nat Struct Biol.

[CR4] Brazma A, Hingamp P, Quackenbush J (2001). Minimum information about a microarray experiment (MIAME) – toward standards for microarray data. Nat Genet.

[CR5] Brazma A, Ball C, Bumgarner R (2012). MINSEQE: minimum information about a high-throughput nucleotide sequencing experiment – a proposal for standards in functional genomic data reporting. Zenodo.

[CR6] Cimini BA, Nørrelykke SF, Louveaux M (2020). The NEUBIAS Gateway: a hub for bioimage analysis methods and materials. Science.

[CR7] Cochrane G, Karsch-Mizrachi I, Nakamura Y, on behalf of the International Nucleotide Sequence Database Collaboration (2011). The International Nucleotide Sequence Database Collaboration. Nucleic Acids Res.

[CR8] Courtot M, Gupta D, Liyanage I (2022). BioSamples database: FAIRer samples metadata to accelerate research data management. Nucleic Acids Res.

[CR9] Cummins C, Ahamed A, Aslam R (2022). The European Nucleotide Archive in 2021. Nucleic Acids Res.

[CR10] Dayhoff MO (1969). Atlas of protein sequence and structure.

[CR11] Ellenberg J, Swedlow JR, Barlow M (2018). A call for public archives for biological image data. Nat Methods.

[CR12] Faas FGA, Avramut MC, van den Berg BM (2012). Virtual nanoscopy: generation of ultra-large high resolution electron microscopy maps. J Cell Biol.

[CR13] Freeberg MA, Fromont LA, D’Altri T (2022). The European Genome-phenome Archive in 2021. Nucleic Acids Res.

[CR14] Gaulton A, Hersey A, Nowotka M (2017). The ChEMBL database in 2017. Nucleic Acids Res.

[CR15] Hammer M, Huisman M, Rigano A (2021). Towards community-driven metadata standards for light microscopy: tiered specifications extending the OME model. Nat Methods.

[CR16] Hartley M, Kleywegt GJ, Patwardhan A (2022). The BioImage Archive – building a home for life-sciences microscopy data. J Mol Biol.

[CR17] Hartwig P, Höglinger D (2021). The glucosylceramide synthase inhibitor PDMP causes lysosomal lipid accumulation and mTOR inactivation. Int J Mol Sci.

[CR18] Haug K, Cochrane K, Nainala VC (2020). MetaboLights: a resource evolving in response to the needs of its scientific community. Nucleic Acids Res.

[CR19] Herrick R, Horton W, Olsen T (2016). XNAT Central: open sourcing imaging research data. Neuroimage.

[CR20] Iudin A, Korir PK, Somasundharam S (2023). EMPIAR: the Electron Microscopy Public Image Archive. Nucleic Acids Res.

[CR21] Jumper J, Evans R, Pritzel A (2021). Highly accurate protein structure prediction with AlphaFold. Nature.

[CR22] Lambert TJ (2019). FPbase: a community-editable fluorescent protein database. Nat Methods.

[CR23] Lawson CL, Patwardhan A, Baker ML (2016). EMDataBank unified data resource for 3DEM. Nucleic Acids Res.

[CR24] Lewis SM, Asselin-Labat M-L, Nguyen Q (2021). Spatial omics and multiplexed imaging to explore cancer biology. Nat Methods.

[CR25] Linkert M, Rueden CT, Allan C (2010). Metadata matters: access to image data in the real world. J Cell Biol.

[CR26] Malone J, Holloway E, Adamusiak T (2010). Modeling sample variables with an Experimental Factor Ontology. Bioinformatics.

[CR27] Marée R, Rollus L, Stévens B (2016). Cytomine: an open-source software for collaborative analysis of whole-slide images. Diagn Pathol.

[CR28] Mendez D, Gaulton A, Bento AP (2019). ChEMBL: towards direct deposition of bioassay data. Nucleic Acids Res.

[CR29] Moore J, Allan C, Besson S (2021). OME-NGFF: a next-generation file format for expanding bioimaging data-access strategies. Nat Methods.

[CR30] Moore J, Basurto-Lozada D, Besson S et al. (2023) OME-Zarr: a cloud-optimized bioimaging file format with international community support. bioRxiv. 10.1101/2023.02.17.528834 10.1007/s00418-023-02209-1 PMC10492740 37428210

[CR31] Moreno P, Fexova S, George N (2022). Expression Atlas update: gene and protein expression in multiple species. Nucleic Acids Res.

[CR32] Peddie CJ, Genoud C, Kreshuk A (2022). Volume electron microscopy. Nat Rev Methods Prim.

[CR33] Perez-Riverol Y, Bai J, Bandla C (2022). The PRIDE database resources in 2022: a hub for mass spectrometry-based proteomics evidences. Nucleic Acids Res.

[CR34] Piwowar HA, Vision TJ (2013). Data reuse and the open data citation advantage. PeerJ.

[CR35] Rayner TF, Rocca-Serra P, Spellman PT (2006). A simple spreadsheet-based, MIAME-supportive format for microarray data: MAGE-TAB. BMC Bioinform.

[CR36] Sarkans U, Gostev M, Athar A (2018). The BioStudies database – one stop shop for all data supporting a life sciences study. Nucleic Acids Res.

[CR37] Sarkans U, Chiu W, Collinson L (2021). REMBI: Recommended Metadata for Biological Images – enabling reuse of microscopy data in biology. Nat Methods.

[CR38] Sartori A, Gatz R, Beck F (2007). Correlative microscopy: bridging the gap between fluorescence light microscopy and cryo-electron tomography. J Struct Biol.

[CR39] Spellman PT, Miller M, Stewart J (2002). Design and implementation of microarray gene expression markup language (MAGE-ML). Genome Biol.

[CR40] Thakur M, Bateman A, Brooksbank C (2023). EMBL’s European Bioinformatics Institute (EMBL-EBI) in 2022. Nucleic Acids Res.

[CR41] The UniProt Consortium (2023). UniProt: the Universal Protein knowledgebase in 2023. Nucleic Acids Res.

[CR42] Varadi M, Anyango S, Deshpande M (2021). AlphaFold Protein Structure Database: massively expanding the structural coverage of protein-sequence space with high-accuracy models. Nucleic Acids Res.

[CR43] von Chamier L, Laine RF, Henriques R (2019). Artificial intelligence for microscopy: what you should know. Biochem Soc Trans.

[CR44] Wilkinson MD, Dumontier M, Aalbersberg IJ (2016). The FAIR Guiding Principles for scientific data management and stewardship. Sci Data.

[CR45] Williams E, Moore J, Li SW (2017). Image Data Resource: a bioimage data integration and publication platform. Nat Methods.

[CR46] wwPDB Consortium (2019). Protein Data Bank: the single global archive for 3D macromolecular structure data. Nucleic Acids Res.

[CR47] Yoshida N, Domart M-C, Peddie CJ (2020). The zebrafish as a novel model for the in vivo study of *Toxoplasma gondii* replication and interaction with macrophages. Dis Model Mech.

[CR48] Zhu X, Zhang Y, Wang Y (2022). Nucleome Browser: an integrative and multimodal data navigation platform for 4D Nucleome. Nat Methods.

